# Variable detours in long-distance migration across ecological barriers and their relation to habitat availability at ground

**DOI:** 10.1002/ece3.1279

**Published:** 2014-10-12

**Authors:** Steffen Hahn, Tamara Emmenegger, Simeon Lisovski, Valentin Amrhein, Pavel Zehtindjiev, Felix Liechti

**Affiliations:** 1 Department of Bird Migration, Swiss Ornithological InstituteSempach, Switzerland; 2 Centre for Integrative Ecology, Deakin UniversityGeelong, Vic., Australia; 3 Zoological Institute, University of BaselBasel, Switzerland; 4 Research Station Petite Camargue AlsacienneSaint-Louis, France; 5 Institute of Biodiversity and Ecosystem Research, Bulgarian Academy of SciencesSofia, Bulgaria

**Keywords:** Distance, geolocator, migration route, optimal migration, time minimization, timing

## Abstract

Migration detours, the spatial deviation from the shortest route, are a widespread phenomenon in migratory species, especially if barriers must be crossed. Moving longer distances causes additional efforts in energy and time, and to be adaptive, this should be counterbalanced by favorable condition en route. We compared migration patterns of nightingales that travelled along different flyways from their European breeding sites to the African nonbreeding sites. We tested for deviations from shortest routes and related the observed and expected routes to the habitat availability at ground during autumn and spring migration. All individuals flew detours of varying extent. Detours were largest and seasonally consistent in western flyway birds, whereas birds on the central and eastern flyways showed less detours during autumn migration, but large detours during spring migration (eastern flyway birds). Neither migration durations nor the time of arrival at destination were related to the lengths of detours. Arrival at the breeding site was nearly synchronous in birds flying different detours. Flying detours increased the potential availability of suitable broad-scale habitats en route only along the western flyway. Habitat availability on observed routes remained similar or even decreased for individuals flying detours on the central or the eastern flyway as compared to shortest routes. Thus, broad-scale habitat distribution may partially explain detour performance, but the weak detour-habitat association along central and eastern flyways suggests that other factors shape detour extent regionally. Prime candidate factors are the distribution of small suitable habitat patches at local scale as well as winds specific for the region and altitude.

## Introduction

A migration strategy might be optimal if travel time, total energy expenditure, or costs of transport are minimized (Alerstam and Lindström 1990; Alerstam 2011) in such a way that the individual arrives at optimal time at the destination, which during prenuptial (spring) migration is prerequisite for high reproductive performance (Moore et al. 2005; Drent 2006). In a hypothetical world with uniform conditions, this could be best achieved by using the shortest distance between departure and destination, if environmental conditions were similar along all potential routes. However, detours, that is, the spatial deviations from the shortest path occur frequently in migratory animals (Alerstam 2001), raising questions about potential underlying factors determining individual migration patterns more than a simple minimization of distance (Alerstam 2011). A detour generally enlarges travel distance, but the cost of such a migration detour might be equal or even smaller than the shortest route: Detours might reduce the risk of predation or disturbance (Klaassen et al. 2006; Ydenberg et al. 2007), can lead to reduced energetic costs if favorable winds assist flight (Erni et al. 2005; Shamoun-Baranes et al. 2010), or reduce the costs in time if stopover sites allow for high fueling rates (Bauer et al. 2010; Lindstrom et al. 2011). The latter highly depends on the availability of suitable habitats with high food availabilities and low risk of predation/disturbance (Pomeroy et al. 2006).

Within the annual cycle of a migratory bird, life history activities like reproduction and molt are scheduled in subsequent periods. The temporal sequences of these events are similar for all individuals within a given species, and hence, the need for their optimal timing is also similar. Consequently, between-population differences in timing and performance of a life history event might be caused by different external environmental conditions like food availability (Studds and Marra 2007; Bauchinger et al. 2009) or flight and weather conditions (Franke et al. 2011), but should not be related to detour extent. Thus, the individuals should be under similar pressure to use optimal migration routes within a particular season, irrespectively of the location of their nonbreeding and/or breeding sites. Moreover, if timing is more crucial, for example, for the onset of reproduction (Perrins 1970; Smith and Moore 2005; Emmenegger et al. 2014), and more relaxed for the arrival at the nonbreeding residence (Conklin et al. 2013), a different selection pressure for using the optimal migration route and schedule can be expected for the spring and autumn migration period.

The energetic costs of migration are mainly determined by total flight distance, length of nonstop flights and its related fuel transport, and the food availability for fueling at the stopover sites (Alerstam and Lindström 1990). However, optimal arrival at final destination is likely to be the evolutionary currency for migrants, which finally determines fitness (Perrins 1970; Kokko 1999). Because fueling influences stopover durations (Marra et al. 1998) and fueling rate might be related to site-specific food availability including predation risk (Lindström 2003; Schmaljohann and Dierschke 2005; Bayly 2007), the distribution of suitable habitats along a migratory route should determine time and costs of a specific journey (Bauer et al. 2010). Hence, a detour encompassing many suitable habitats for stopping over might have the same significance for optimal timing as choosing the shortest route offering limited possibilities for fueling. This might lead to different expectations: Detours should include areas with suitable habitats, especially if a detour significantly enlarges the particular migration distance. Further, as almost all habitats are seasonal in temperate regions, detours can differ seasonally, which might be co-affected by seasonally different needs for optimal timing of the journey, for example, arrival at breeding site and at the nonbreeding residence site.

The western Palaearctic-African migration system is highly suitable for studying detours in migrating landbirds, because potential fueling habitats as well as migration barriers, for example, sea, desert, and high mountain ridges, are arranged in latitudinal bands of different size in west-eastern direction. Additionally, distant breeding populations of the same species often differ substantially in their main migration direction (e.g., Zink and Bairlein 1995). Because all migrants must finally move south to enter sub-Saharan Africa (and vice versa), any detours are deviations in longitude. Moreover, land mass and the distribution of fueling habitats allow for wide longitudinal detours to circumvent the ridge of the Alps and the Mediterranean Sea.

**Figure 1:**
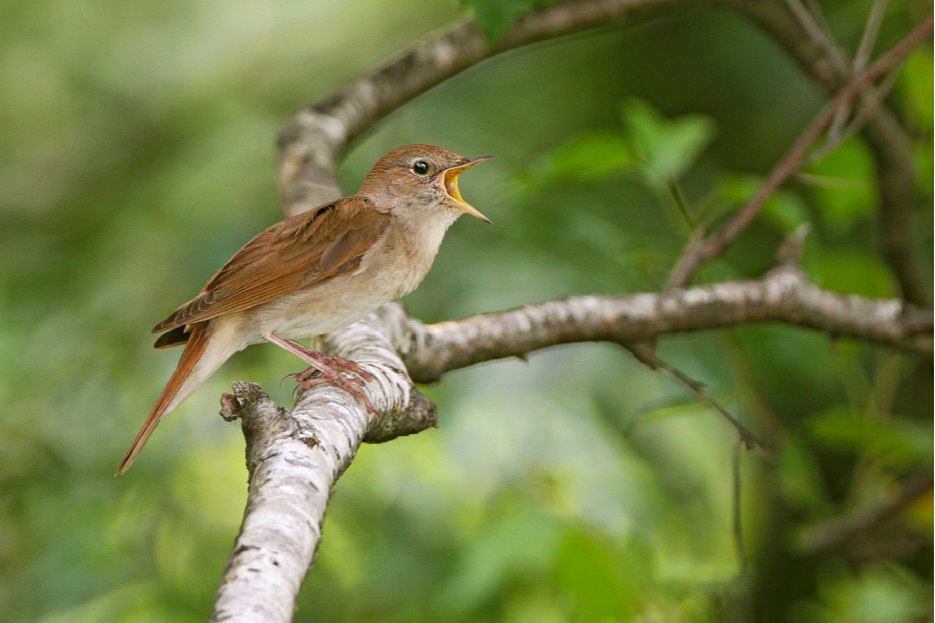
The common nightingale *Luscinia megarhynchos* is a typical Palearctic woodland species who spends the nonbreeding season in sub-Saharan Africa.

We analyzed migration detours in long-distance migrating adult common nightingales *Luscinia megarhynchos* (Fig. 1) from three European breeding populations that use the south-western, southern, or south-eastern flyway toward Africa (Korner-Nievergelt et al. 2012). All individuals were confronted with geographical barriers on their routes, that is, the Mediterranean Sea and the Sahara desert, which they might circumvent along their edges or cross at the narrowest point to minimize inhospitable conditions at ground. We tracked birds older than 1 year that already did the journey at least once before, to record individual routes along a chain of stopover sites (Mouritsen 2003). Individuals from all populations share a preference for densely vegetated habitats like woodlands, forest edges, and shrubs during breeding, nonbreeding (Moreau 1972; Zwarts et al. 2009), and presumably during migration. In addition to similar habitat preferences, the geographically separated populations share the evolutionary pressure regarding optimal timing of migration for a timely arrival at the breeding sites to match the local spring green-up (Emmenegger et al. 2014).

Accordingly, we hypothesized that (1) all birds perform detour flights, because all shortest routes included barrier crossings of various extents. Moreover, we expected (2) seasonally different extents and durations of detours, with shorter distances and duration in spring due to strong selection for optimal arrival for breeding. Finally, we hypothesized that (3) detours are generally associated with more suitable habitats for fueling for a woodland species, than are found along the shortest migration route.

## Materials and Methods

### Study system

We studied migration patterns of common nightingales from a western European population in France (47.6°N, 7.5°E; north of the Alps), from a central European population in Italy (44.6°N, 11.8°E; south of the Alps), and from an eastern European population in Bulgaria (with two subsites: 42.1°N, 27.9°E and 43.4°N, 28.3°E). The western and central breeding populations were geographically separated by the Alps, and the eastern population was in about 1300 km distance from the nearest (central) population. According to ring recoveries, these populations are representative of birds using the western, central, and eastern flyways for crossing the Mediterranean Sea and Sahara desert (Korner-Nievergelt et al. 2012) and are labeled accordingly.

We equipped 100 adult nightingales (63% males) per site with geolocators in April to June 2009 using mist nets for capturing breeding birds in their territories. Geolocators (SOI-GDL1.0; Swiss Ornithological Institute; mean mass: 1.12 g including harness =4.8% of average adult body mass) were attached to the bird's back using a leg-loop harness made from flexible silicone. To retrieve geolocators in 2010, we recaptured birds in their previous breeding territories and in surrounding areas. Recapture rate varied between sexes and study sites, with an average of 26% for males and 8% for females (for details see Hahn et al. 2013). After loss or malfunctioning of some geolocators, we obtained data from 28 individuals (11 from the western, six from the central, and 11 from the eastern population) on autumn migration and nonbreeding sites and 11 individuals on spring migration (5, 2, and 4 birds from the western, central, and eastern population).

### Data processing

We used the light level threshold method for positioning. Sunset and sunrise were determined from daily light measurements of 5-min interval. Geolocator data analysis including calculation of positions was carried out with the R package GeoLight (1.02) in R (Lisovski and Hahn 2012). We determined main stationary periods from movement periods for each bird using similarities in subsequent sun events (function ChangeLight with 90% quantile threshold probability and a minimum staging period of 3 days). Changes during the nonbreeding period were additionally detected on a smaller scale (ChangeLight with 70% quantile threshold probability). Outliers within stationary periods were removed before further processing using the function distance filter with a threshold average flight speed of 30 km·h^−1^ (see R package manual for function details, Lisovski and Hahn 2012). We merged subsequent periods if positions were at the same site (excl. periods during equinox times).

We applied period-specific approaches to calibrate data of stationary periods due to seasonally different shading conditions by variable environment and/or bird's behavior. For nonbreeding sites, we used the Hill–Ekstrom calibration method, that is, the variance minimization of latitude (Lisovski et al. 2012). The resulting sun elevation angles ranged between −6° and −1.3° (mean: −4.5°). However, the core requirement for Hill–Ekstrom calibration, that is, invariable shading intensity within a focal period was not fulfilled for the staging periods during both migration legs. Thus, we used individual sun elevation angles derived from measurements on bird at their breeding sites before departure in July for autumn migration and after arrival in April for spring migration (in-habitat calibration, mean sun elevation angles for autumn: −4.2°, for spring: −5.1°).

We defined “nonbreeding sites” as areas of residency in sub-Saharan Africa during the nonbreeding season. Staging sites are sites occupied for short periods of time during migration between breeding and the main nonbreeding site. To delimit specific sites, we used the polygon encompassing 70% of kernel density (ESRI ArcGIS 9.3, kernel density analyses with search radius of 300 km).

### Detours and the shortest migration route

We compared realized (observed) migration routes in autumn and spring with the shortest migration route defined as the loxodromic distance (i.e., the path with constant compass bearing) between breeding site and nonbreeding site for each individual. As location of nonbreeding residences, we used the centroid points of kernel density polygons. Loxodromic distances differed from great circle distances by 0.13% (range: 0.02–0.34%). If an individual occupied several sites of nonbreeding residency in succession, the expected shortest migration routes were separately calculated for the earliest occupied nonbreeding site after autumn migration and for the latest site before spring migration.

Nightingales mainly migrated during equinox periods (September/October and March/April), in which latitude could not be determined by threshold-based geolocation (Lisovski et al. 2012). We therefore focused on relative longitudinal position in relation to the shortest route, and we thus rescaled the time of the journey from departure until arrival to 100. For each observed longitudinal position at its relative time, we calculated the deviation from the corresponding longitude of the shortest route. This procedure provides continuous information on relative positions in relation to shortest route but ignores stopovers by assuming a continuous movement.

We categorized the migrants according to the detour pattern from their individual shortest migration route, that is, the deviation from “optimal” longitude, the occupied detour sector and the relative time period at a specific sector (Table 1). Sectors were defined as the optimal sector if deviations ranged between −1° and 1° longitude (similar range in-habitat modeling, see below), the western detour sector with deviations ≤−1° and the eastern detour sector with deviations ≥1° longitude. We considered an exclusive sector use if ≥80% of daily means of longitude deviations per individual fell in the focal sector (western, eastern detour, or the shortest route). A mixed detour applied if the bird used a sector by 50–79% of time plus the neighboring sector(s). A detour switch occurred if the bird leaped from the eastern to the western detour sector (and vice versa) without being longer times at the intermittent optimal sector.

### Habitat modeling

We extracted habitat types along observed and shortest routes from Global Land Cover 2000 data set (http://bioval.jrc.ec.europa.eu/products/glc2000/data_access.php). Habitats were categorized according to their potential suitability for foraging by insectivorous nightingales: We classified forests (habitat with dominant tree cover), semi-open habitats (cropland, shrubs with trees, cultivated managed areas, irrigated agriculture), and open habitats (sparse herbaceous and sparse shrub cover) as suitable habitats. Nonsuitable habitats were areas without vegetation (desert, ice cover, and urban areas) and water. For shortest routes, we selected habitats from polygons with edges of 1° west and east from the shortest migration route. Data were log-transformed for statistical tests (repeated measure (RM) ANOVA). The polygons for observed migration routes were formed by the envelope from the breeding site and the individual nonbreeding residence (±1° to avoid an arrow shape toward the nonbreeding site) and all staging sites of all individuals within the same detour category (in relation to shortest route, see above). Hence, habitat polygons for observed routes per detour category share edges for breeding and staging sites but not for individual nonbreeding residences (Fig. S1).

## Results

### General spatial migration pattern

**Figure 2:**
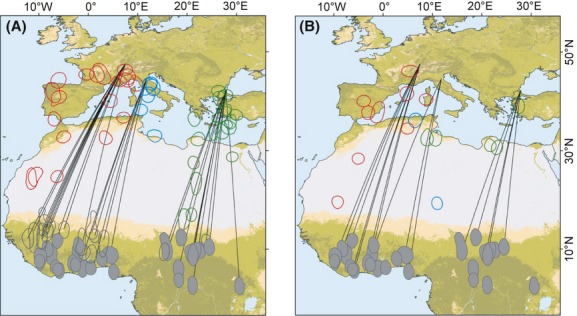
Stationary sites of nightingales from three populations during autumn (A) and spring migration (B). Stopover sites are given in red (western population), blue (central), and green (eastern population), and nonbreeding residence sites are filled gray polygons. 61% of birds used a prewinter site (open gray polygons) in sub-Saharan Africa for 38 ± 10.5 days before arriving at the final winter destination. Shortest routes for autumn and spring are given as black lines; map colors indicates the five habitat categories with forests (dark green), semi-open habitats (light green), open habitats (yellow) as suitable habitats, and areas without vegetation (gray) and water (blue) as nonsuitable habitats for nightingales.

During autumn migration, almost all birds used the expected population-specific main migratory flyway: For the western population, 8 of 11 individuals (73%) migrated westwards via the Iberian Peninsula and across the western Mediterranean islands, but three individuals migrated SSW closely to the central flyway, crossing the Mediterranean Sea toward the eastern Atlas Mountains (Fig. 2A). Birds from the central population crossed the central Mediterranean, and birds from the eastern population crossed the Mediterranean at south-western Turkey, Aegean Sea, and Crete (Fig. 2A). In spring, birds from the western and central populations mainly followed the same patterns as during autumn. However, two of five birds from the eastern population used the central flyway in spring, while the other three individuals used the eastern flyway (Fig. 2B).

Seventeen of 28 birds (61%) used more than one nonbreeding residence in Africa, which caused seasonally different shortest loxodromic migration routes. Proportions of birds with multiple nonbreeding sites differed between populations, with 91% and 83% in the western and central populations but only in 18% in the eastern population (Fig. 2A). Mean distance between the first nonbreeding site after desert crossing and the main nonbreeding residence averaged at 590 ± 266 km and 4.4° toward the equator (largest difference in the western population: 655 ± 293 km).

### Detours and spatial patterns of migration

**Figure 3:**
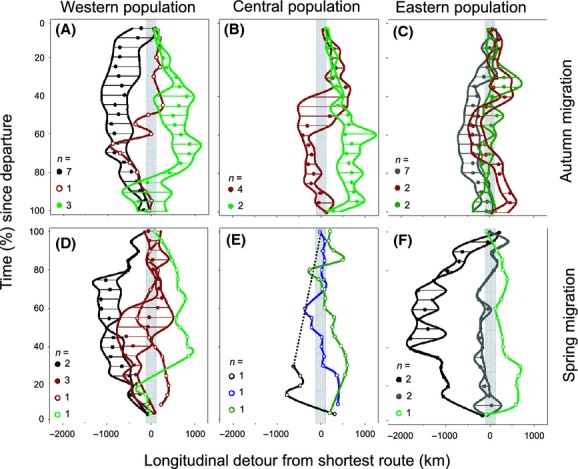
Longitudinal detours (in km) from the shortest route during autumn (A–C) and spring migration (D–F) observed in common nightingales. The shortest longitudinal route sector (gray) is derived from the loxodromic route ± 1° between destinations. Data are grouped in categories of 5% of time (since departure); lines within the envelopes indicate 25–75% percentiles of longitude detours, dots give medians, and sample size per category is indicated within each panel. Detour categories are symbolized in black and green for western and eastern detours, dark gray, and dark green are detours mainly on western and eastern sectors incl. changes toward the loxodromic sector, and red are switches between western and eastern detour sectors.

All birds flew detours to some extent, and individuals from the same population did not show a uniform preference for the loxodromic or one of the detour sectors (Fig. 3, Table S1). Moreover, 9–33% and 57% of migrants, respectively, switched between a western detour and an eastern detour during autumn and spring migration (Fig. 3). Detours during autumn migration, that is, absolute deviations from the shortest route, were largest in the western population and smallest in the eastern population (Linear mixed effect model (LMM) for detour differences with bird ID as random factor: west vs. east: *t* = −3.87, *P* = 0.001, all other: *P* > 0.05, Fig. 3A–C). Detours during spring migration did not differ between populations (LMM, all *P* > 0.37), probably due to the small sample size of tracked individuals in spring (Fig. 3D–F).

The individuals of the western population that flew western detours (Fig. 3A+D) used staging sites in western France, Spain, Portugal, and the western Atlas mountains; eastern detour sector birds stoppedover in south-eastern France, along the coast of Liguria (Italy), and the eastern Atlas (Fig. 2). Birds from the central population mainly made eastern detours (Fig. 3B+E), and staging site were at the Apennine peninsula, the eastern Atlas, and the Libyan coast (Fig. 2). Birds from the eastern population made relatively small detours toward west and east in autumn (Fig. 3C), but in spring, two of five individuals performed wide western detours with maximal deviations of 14° and 18° toward west (Fig. 3F). Thus, birds stopped over at sites in the Aegean archipelago, in oasis of the eastern Sahara (Fig. 2A) and, as the most extreme deviation, at the coast of Cyrenaica and Tunisia (spring migration only, Fig. 2B).

**Figure 4:**
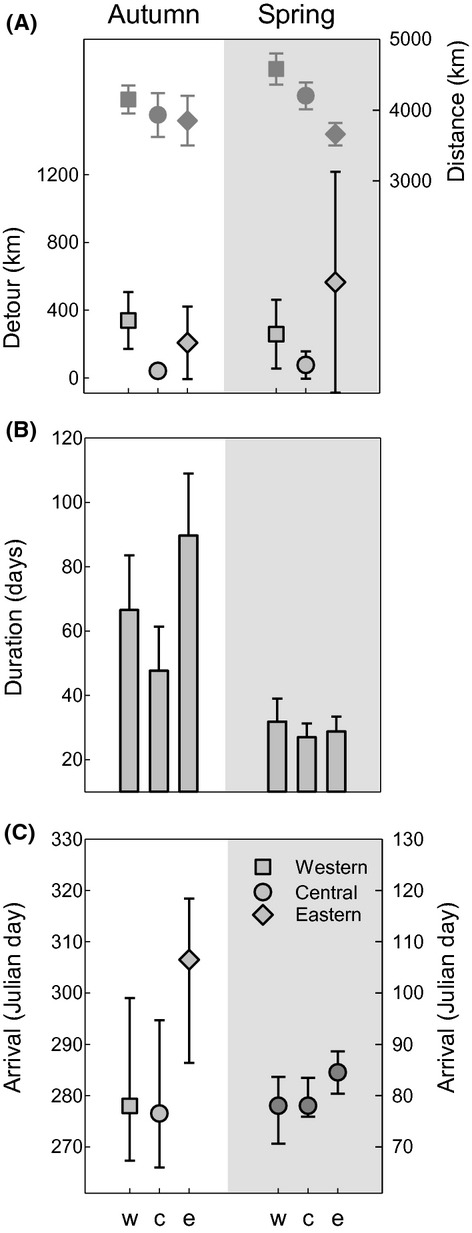
Main measures of distance, duration, and arrival for autumn migration (left) and spring migration (right) of nightingales from western (square), central (circle), and eastern breeding populations (diamond). (A) gives average shortest distances between departure and arrival destinations and observed detour distances (km), (B) average duration of the particular migration leg and (C) average arrival date at wintering and breeding sites (day 1 = 1 Jan). All measures are population-specific means ± SD.

The expected shortest migration distance varied between 3400 km and 4500 km on autumn migration and between 3400 km and 4800 km on spring migration, with longest routes for the western population (ANOVA population differences in autumn: *F*_2,28_ = 3.17, *P* = 0.06, but test power = 0.05; in spring: *F*_2,28_ = 15.6, *P* = 0.001; Fig. 4A). The difference between detour distance and expected shortest route distance were population specific (factor population: *F*_2,28_ = 3.7, *P* = 0.04, Fig. 4A) with similar patterns for autumn and spring migration, respectively (factor season: *F*_1,28_ = 2.05, *P* = 0.17). Birds from the central population consistently showed smallest detour distance (Tamhane post hoc tests on differences to the other two populations: both *P* = 0.01; Fig. 4A).

### Detours and temporal patterns of migration

Birds departed from breeding areas on average during the first week of August in the western and eastern populations and two weeks later in the central population (ANOVA: *F*_2,27_ = 13.1, *P* = 0.001; pairwise comparison west and east vs. central *P* < 0.05). Birds of various detour categories (Table S1) did not depart at different times (calculated as deviations from site-specific mean: *F*_2,27_ = 0.61, *P* = 0.55). Birds arrived at their nonbreeding residences in the second week of October (western and central population) or in the last week of October (eastern population) (*F*_2,26_ = 3.41, *P* = 0.05, west vs. east *P* = 0.05, Fig. 4C). Departure dates from the nonbreeding residence were rather similar across populations (mean: 21 March ± 5.8 SD; *F*_2,15_ = 3.10, *P* = 0.08; Fig. 4C). Moreover, departure dates in the eastern population did not differ between the two birds using the extreme western detour (22th and 26th March) and the three other birds (20th, 25th and 29th March). Finally, birds arrived almost synchronously at their breeding sites (mean: 19 April ± 5.3 SD; population difference *F*_2,13_ = 0.86, *P* = 0.45).

The average duration of autumn migration of 73.3 days ± 2 SD was significantly longer than the spring migration with 30.1 days ± 5.9 SD (for periods: *F*_1,42_ = 43.21, *P* = 0.001, interaction term population × period: *F*_2,42_ = 2.88, *P* = 0.07, Fig. 4B). There was no relationship between duration and observed distance during autumn migration (reduced major axis regressions: *R*^2^ = 0.01, *n* = 28, Fig. S2) or during spring migration (reduced major axis regressions: *R*^2^ = 0.05, *n* = 14, Fig. S2).

### Detours and habitat association

**Figure 5:**
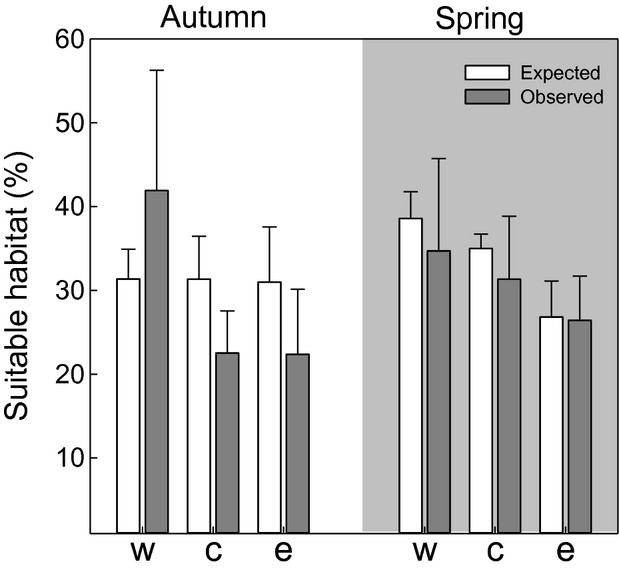
Habitat composition along expected shortest routes and observed routes during autumn (left) and spring migration (right) of common nightingales. Suitable habitats encompass forests; semi-open and open habitats (see methods for habitat categories, Fig. 1 for continental distribution). Bars give mean percentage (±SD) for birds from western (w), central (c) and eastern population (e).

The expected composition of suitable habitats, that is, forests, semi-open, and open habitats along shortest routes were similar for all populations, with 31% suitable habitats in autumn (*χ*^2^ = 6.081, df = 4, *P* = 0.19, Fig. 5) and 34% suitable habitats in spring (*χ*^2^ = 6.09, df = 4, *P* = 0.19) (Fig. 5). The nonsuitable habitats contributed to about 47–50% (desert) and 19% (water) of shortest route habitats for autumn and spring. Generally, the availability of suitable habitats differed between detour and expected shortest route (repeated measures (RM) ANOVA *F*_1,55_ = 8.13, *P* = 0.01) with flyway-specific pattern (RM ANOVA interaction population × detour *F*_2,55_ = 14.20, *P* < 0.001). For autumn migrants along the western flyway, we found a significantly higher proportion of suitable habitats along the detour route (42%) compared to the expected shortest route (31%) (Holm–Sidak post hoc test: *t* = 2.68, *P* = 0.01), mainly caused by semi-open habitat availabilities (expected: 12%, detour: 22%, Fig. 5). In contrast, autumn detour routes of birds from the central and the eastern population comprised only 22.5% of suitable en route habitats, which is a significant reduction compared to shortest route habitat composition (Holm–Sidak post hoc test: for the central population: *t* = −2.99; *P* = 0.006; for eastern population: *t* = 4.22, *P* = 0.001; Fig. 5).

In spring, habitat composition along detour routes did not differ from those along the expected shortest routes in all populations (RM ANOVA factor route: *F*_1,29_ = 2.29, *P* = 0.16; interaction habitat suitability × population: *F*_2,29_ = 0.41, *P* = 0.68) (Fig. 5). However, birds from the western population had more suitable habitats along their flyway than birds of the eastern population (RM ANOVA, factor population: *F*_2,29_ = 5.33, *P* = 0.02, pairwise comparison: west vs. east *t* = 3.24, *P* = 0.02, all others *P* > 0.05), (Fig. 5).

## Discussion

### Detours and spatial-temporal migration pattern

Detours in (bird) migration are a widespread phenomenon in various guilds including herbivores (fig. 3 in Green et al. 2002), insectivorous ground feeders (figs 1 in Schmaljohann et al. 2012; and in Tottrup et al. 2012b), insectivorous aerial feeders (fig. 1 in Akesson et al. 2012), or waders (fig. 1 in Lindstrom et al. 2011). However, detours are often found if complete migration legs over large distances and across various habitats are considered whereas shortest distance flights (great circle routes) are recorded for specific parts within a migration leg and often in high northern latitude, where diversity of habitats might be reduced (Alerstam et al. 2001, 2007, 2008). Here, we verify a heterogeneous pattern in migration detours in three nightingale populations with wider detours in western compared to eastern populations but little seasonal variation in detour magnitude.

The predominant western direction of detours for the western population might be explained by the general distribution of landmasses (Fig. 2), but this simple explanation is not valid for birds of the central and eastern population that crossed the Mediterranean Sea instead of flying eastwards overland across the Near East (the eastern population). Consequently, the route-shaping factors must be geographically distinct (Erni et al. 2005). Furthermore, detour lengths and detour directions varied between simultaneously tracked individuals within a population indicating that multiple routes potentially provide suitable conditions to reach the final destination (Bauer et al. 2010). High variability in migration routes within a local population seems to be a common case in many species and encompasses variation between individuals (e.g., Akesson et al. 2012; Tottrup et al. 2012b; Kristensen et al. 2013; Ross et al. 2014; this study) as well as between-year variation within the same individual (e.g., Vardanis et al. 2011; Stanley et al. 2012). Different migration routes with their specific environmental conditions may in turn contribute to the population-specific variation in arrival times, body condition upon arrival, or even in the onset of reproduction (e.g., Purcell and Brodin 2007).

In contrast to the prediction of smaller detours in spring compared to autumn migration due to seasonally different migratory constraints, birds showed either a very similar migration pattern in both seasons or, in the eastern population, two individuals on spring migration even made the largest detours recorded within the study. Thus, total migration distances covered in spring were not reduced but even longer than the corresponding autumn migration distances, which could result in similar or longer total migration durations. This would contrast the expectations for a time minimization strategy during spring migration in general (see also Nilsson et al. 2013); however, we did not find a positive relation between total migration duration and detour extent within a season and between autumn and spring migration, and in fact, the spring journey was about two times faster than the autumn journey.

To achieve a similar or shorter migration duration over a longer distance means either higher migration speed (air speed), wind assistance (higher ground speed), shorter stopover times, or a combination of these factors. There is theoretical and empirical evidence that the summed length of stopover times overrides the effects acting during the movement periods (e.g., Alerstam and Lindström 1990; Houston 2000; Nilsson et al. 2013). Hence, we suggest that birds using different detours differ in their stopover durations and consequently in site-specific fueling rates.

In spring, a timely (early) arrival on the breeding grounds requires fast spring migration, which can only be achieved by efficient refueling on intermittent stopover sites and might be facilitated by high food availability on these sites. In contrast, during autumn migration such time-constraints might not exist, and a safe arrival in the nonbreeding grounds might be more important than a timely arrival. Here, a route with a lower predation risk might be the better choice. Variable detours during different migrations might therefore indicate that individuals value variables along the route differently depending on the season.

To fly a detour might be facilitated by assistant wind conditions for energetically cheap and/or fast flights. The departure from a stopover site in relation to wind conditions aloft can impact the distance flown with a given amount of fuel (Weber et al. 1998). Thus, wind support can lower the costs of flight and consequently may reduce stopover times for refueling to some extent. For Palaearctic-African migrants, wind assistance seems important for desert crossing in autumn (Erni et al. 2005; Barboutis et al. 2011) and songbirds crossing the Sahara actively choose altitudes with favorable winds, and thereby altering flight altitudes between 500 to 5000 m.a.s.l. (Schmaljohann et al. 2009). Thus, modeling wind support for an individual migrating songbird would require accurate spatial and altitudinal positions, and this is still far beyond the potential of geolocation by light, especially during the equinox periods. However, wind directions at a broad scale might be similar for birds using the same flyway at the same time, but they might not fully explain the highly different detours we found within a focal population along the same flyway (i.e., Fig. 3D+F). Nilsson et al. (2013) recently showed that flight speeds (ground and air speeds) did not differ between season in a similar extent than stopover duration and total duration of migration. Consequently, we argue that stopover conditions for fueling and thus habitat might be a most important driver for the selection of individual migration routes.

### Detours and habitat association

All migrants have to fuel for their migratory flight, especially if large barriers like the Sahara desert must be crossed (e.g., Biebach et al. 1986; Barboutis et al. 2011). Taking the longer distance of a detour seems beneficial if favorable stopover sites allowing for high fueling rates are available, and thus, detouring birds could profit from short stopovers and might be in good condition at arrival for subsequent performance (Alves et al. 2012). Detours in nightingales along the western flyway increased flight overland (Europe), decreased sea crossing and allowed for stopovers in NW Africa and the Atlas Mountains, which likely have similar good food availabilities as the southern Iberian Peninsula. Hence, western migrants might have followed habitat availability at a broader scale, that is, in the landscape context (Buler et al. 2007; Ktitorov et al. 2008; Buler and Moore 2011). The NW African stopover sites (and wind assistances for subsequent Sahara crossing) seems fundamental for high survival rates in south-westerly migrating passerines during autumn (Erni et al. 2005). However, detours performed by birds from the central and eastern population could not be explained by our broad-scale habitat approach. This may point toward stopover habitats on a much smaller scale than we could detect using geolocation.

Threshold-based geolocation does not allow for latitude estimates during equinox periods (Lisovski et al. 2012), the main migration period in nightingales. Further, accuracy in geolocation is fundamentally affected by shading during sunset and sunrise and positioning of woodland species like nightingales are particularly prone for inaccuracy (Lisovski et al. 2012). Stopover site locations near equinoxes contain larger inaccuracies than winter site estimates, and the realized migration distances must be seen as minimum distances, because equinox stopover sites are missed. However, we think that our approach is suitable for detecting route-habitat associations on a broad scale, when habitat patches are as large as along the route of the western population, in comparison with more local scale patches of, for example, small oasis in the desert along the central and eastern flyways.

Our findings of similar spatial detour extents but different temporal detour patterns points toward seasonally different behavior of individuals at staging sites with shorter stops and higher fueling rates in spring than in autumn (see also Nilsson et al. 2013). Surprisingly, the much faster spring migration compared to autumn, was not due to shorter routes, but must have been driven by other factors like faster fueling or possibly by more favorable wind conditions. Moreover, the persistence and the actual condition at a particular staging site might be more critical during spring than during autumn migration. Individuals using isolated stopover sites, as probably along the central and eastern flyway, are particularly vulnerable to habitat deterioration and habitat loss that can immediately affect migration performance (Tottrup et al. 2012a) and thus, might carry-over to subsequent reproduction performance.

## Supporting Information

**Table S1.** Population-specific detour categories during autumn and spring migration in common nightingales *Luscinia megarhynchos*. **Figure S1.** Contour polygons for modelling individual habitat availability along observed migration routes. **Figure S2.** Relationship between observed migration distance and the duration of autumn migration (left) and spring migration (right) in common nightingales.
