# Supplementary material for: Variable detours in long-distance migration across ecological barriers and their relation to habitat availability at ground
Source: Ecol Evol. 2014 Oct 12;4(21):4150–60. doi: 10.1002/ece3.1279 (PMC4242566; doi:10.1002/ece3.1279)
Supplement: Supplementary file 1 [file ece30004-4150-SD1.docx]

**Electronic Supplemental Material (Hahn et al., Detours in Migration)**

Table S1: Population-specific detour categories during autumn and spring migration in common nightingales *Luscinia megarhynchos*. Changes between two adjacent detour sectors or switches between western and eastern detour sectors occurred frequently. The detour sectors are West (< -1° deviation longitude), East (> -1° deviation longitude), and LOX indicates the optimal loxodromic route sector (deviation longitude between -1° and 1° from the loxodromic route). Switches (incl. direction) are leaps between the boundary sectors.

| Population | Season | Detour category | | | | | | n |
| --- | --- | --- | --- | --- | --- | --- | --- | --- |
|  |  | West | West-LOX | LOX | LOX-East | East | Switch West-East |  |
| Western | Autumn | 7 |  |  |  | 3 | 1 (East-West) | 11 |
|  | Spring | 2 |  |  |  | 1 | 3 (West-East), 1 (East-West) | 7 |
| Central | Autumn |  |  |  |  | 4 | 2 (East-West) | 6 |
|  | Spring | 1 |  | 1 | 1 |  |  | 3 |
| Eastern | Autumn |  | 7 |  | 2 |  | 2 (East-West) | 11 |
|  | Spring | 2 | 2 |  |  | 1 |  | 5 |

1

ESM Fig. 1


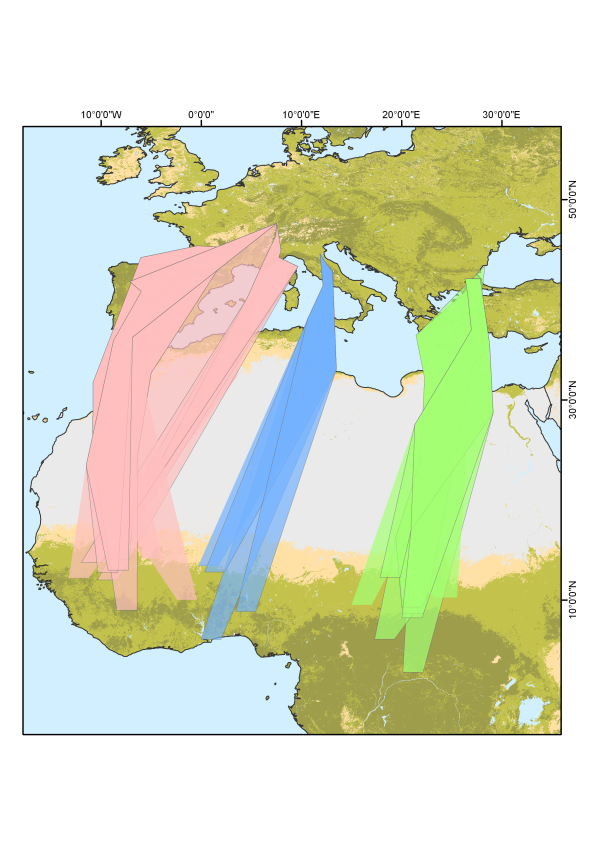

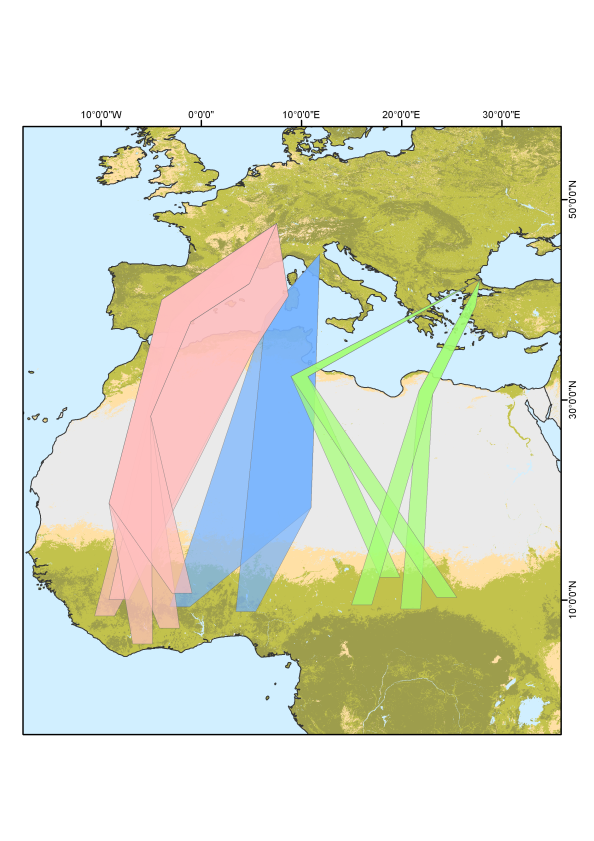


(a)

(b)

10°W

0°

10°E

20°E

30°E

10°W

0°

10°E

20°E

30°E

30°N

50°N

10°N

Figure S1: Contour polygons for modelling individual habitat availability along observed migration routes. Light red for western, blue for central and green for eastern population during (a) autumn migration and (b) spring migration. Formation of polygons based on breeding and non-breeding residence sites and specific stopover sites determined by geolocation (see main text for details).

ESM Fig. 2

Fig. S2: Relationship between observed migration distance and the duration of autumn migration (left) and spring migration (right) in common nightingales. Birds from different populations are symbolized by squares (western population), circle (central population) and diamond (eastern population). The observed distance is the minimum distance covered by each individual (see methods for details).
